# Role of *CD135/CD117* on Prognosis and Overall Survival of Acute Myeloid Leukemia

**DOI:** 10.31557/APJCP.2019.20.9.2625

**Published:** 2019

**Authors:** Mortaza Raeisi, Ali Reza Nikhanfar, Babak Nejate, Ali Akabr Movassaghpour Akbari, Roya Dolatkhah, Yousef Roosta, Zohreh Sanaat

**Affiliations:** 1 *Tabriz University of Medical Sciences, *; 2 *Hematology and Oncology Research Center, Tabriz University of Medical Sciences, Tabriz, Iran. *

**Keywords:** Acute myeloid leukemia, *CD135*, *CD117*, flow, cytometry

## Abstract

**Background:**

The key proliferative RTKs for AML include c-KIT receptor (*CD117*) and FLT-3 receptor (*CD135*). The aim of this study was to evaluate the *CD135* and *CD117* expression, co-expression of *CD135* and* CD117* (*CD135*+*CD117*), and the association of that co-expression with Event Free Survival (DFS) and Overall Survival (OS) rates.

**Material and Methods:**

We analyzed *CD117* and *CD135* expression on AML blasts by flow cytometry and its association with Event Free Survival (DFS) and Overall Survival (OS) in 66 AML treated on Hematology-Oncology Research Center, Iran, Tabriz.

**Results:**

The overall OS and EFS were 50% and 80.3% respectively during our study. Cox-regression analysis revealed that a poor EFS was significantly associated a low *CD135* (HR 0.34, 95% CI 0.13–0.88, P = 0.02).

**Conclusion:**

This is the first study from Iran to show that the expressions of *CD135*, *CD117* is easily measurable by routine diagnostic flow-cytometry, and *CD135*+*117* were not significantly associated with CR, EFS, or OS .

## Introduction

Mutations in multiple genes and improper expressions by epigenetic abnormalities are common findings in most malignancies. A multi-stage mutation process has been posited in cancer research, with evidence of a combination of uncontrolled growth (oncogenes) and dysregulation of growth inhibition (tumor suppressor genes) (Kinzler and Vogelstein, 1997; Malaise et al., 2009). The tyrosine kinase receptors (RTKs) are a family of proteins that have a transmembrane domain and a tyrosine kinase motif (Muller-Tidow et al., 2004; Robinson et al., 2000). These act in cell signaling for the control of growth, differentiation, adhesion, migration, and apoptosis (Hubbard and Till, 2000; Muller-Tidow et al., 2004). Together with downstream effectors, RTKs are known to be key elements in the molecular pathogenesis of malignancy (Malaise et al., 2009). Specifically, mutations in RTKs have been identified in disorders such as myelodysplastic syndrome and acute myeloid leukemia (AML) (Malaise et al., 2009). Among these, AML is the most common leukemia in adults and continues to have the lowest survival rate of all leukemias (Carow et al., 1996; Linnekin, 1999; Sharawat et al., 2015). It is a heterogeneous clonal disease of haemopoietic progenitor cells (“blasts”) that lose the ability to differentiate and proliferate (Malaise et al., 2009). Moreover, AML accounts for 1.1% of all new cancer cases in the USA, has a incidence of 4.3 per 100,000 men and women per year, and accounts for 2.8 deaths per 100,000 men and women per year. The disease is most frequently diagnosed among people aged 65–74 years (“Cancer Stat Facts: Leukemia - Acute Myeloid Leukemia (AML),”) (“Cancer Stat Facts: Leukemia - Acute Myeloid Leukemia (AML),”).

The pathogenesis of AML involves an imbalance between proliferation and apoptosis. RTKs contribute significantly to leukemogenesis, (Sharawat et al., 2013) with the stem cell factor receptor (c-Kit or *CD117*) and FMS-like kinase-3 (FLT3 or *CD135*) being key proliferative RTKs in AML (Sharawat et al., 2013). Both *CD117* and *CD135* are members of the class III RTK family and share the common structure of five extracellular immunoglobulin-like domains, a single transmembrane segment, a juxtamembrane domain, and a split cytoplasmic domain (Masson and Ronnstrand, 2009; Noronha et al., 2016). CD117 is a 145-kD tyrosine kinase transmembrane receptor that is expressed in 4% of normal bone marrow mononuclear cells, including stem cells, progenitor cells, and mast cells (Ashman,et al., 1991; Wells et al., 1996). It is also a diagnostic marker of AML that is expressed in 85% of affected patients, (Bene et al., 1998; Sharawat et al., 2013) and reports are unclear as to whether overexpression is related to outcomes (Ashman et al., 1988; Reuss-Borst et al., 1994; Sharawat et al., 2013). In addition, *CD135* overexpression is associated with poor results in AML (Graf et al., 2004). Given that both *CD117* and *CD135 *are proliferative markers, increased expression of the markers indicates an increased proliferation index, which is known to be important to the pathophysiology of AML (Sharawat et al., 2013). To date, however, no data have been reported on the combined expression of both these proliferative markers, and there has been no evaluation of how they are related to the outcomes of AML.

The aim of this study was to evaluate the frequencies of *CD135* and *CD117* expressions in leukemic blast cells, the degree of *CD135* and *CD117* co-expression (*CD135*+*CD117*), and the association of that co-expression with the event-free survival (EFS) and overall survival (OS) rates.

## Materials and Methods

*Patient selection, treatment, and sampling*

We enrolled consecutive patients with newly diagnosed de novo AML between September 2016 and December 2017 from the Hematology-Oncology Research Center, Iran, Tabriz. The study protocol was approved by the ethics committee of Tabriz University of Medical Sciences (Permit no 5/d/85187) and informed consent was obtained from participants to evaluate peripheral blood and/or bone marrow samples for the study. Patients with acute promyelocytic leukemia and acute lymphoblastic leukemia (ALL) were excluded.

*Diagnosis and treatment*

All diagnoses were made by the following techniques: (1) standard morphology and cytochemistry of peripheral blood and bone marrow films according to the French–American–British criteria (Newell et al., 2003) (before morphologic evaluation, staining was done with Wright’s stain and hematoxylin and eosin); and (2) immunophenotyping with a comprehensive panel of monoclonal antibodies against myeloid- and lymphoid-associated antigens, as proposed by the European Group for the Immunological Characterization of Leukemias (Bene et al., 1998; Li et al., 2008; Malaise et al., 2009). 

A uniform treatment protocol was followed for all patients. This involved induction with a 3+7 regimen (daunorubicin 45 mg/m^2^ and cytosine arabinoside 100 mg/m^2^) followed by two cycles of a 5+2 protocol after remission (daunorubicin 45 mg/m^2^ for 2 days, plus cytosine arabinoside 100 mg/m^2^ for another 5 days). Patients who were not in complete remission (CR) morphologically after the first session of induction chemotherapy received cytosine arabinoside 500 mg/m^2^ by slow intravenous push twice a day for 7 days and Novantrone 12 mg/m^2^ daily for 3 days.

*Flowcytometry*

**Figure 1:**
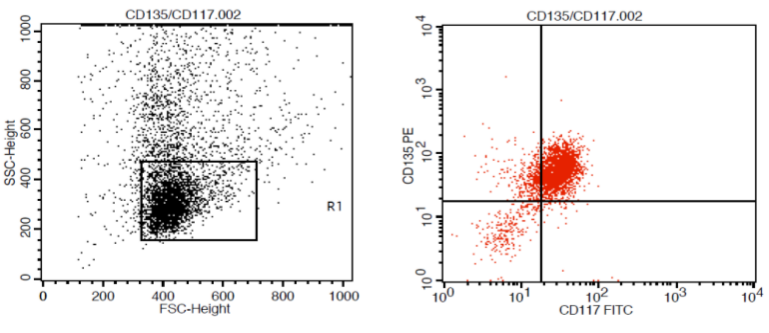
Flow Cytometric Based Coexpression of *CD135*+*CD117*

We obtained 5 mL of peripheral blood (if peripheral blats ≥30%) and bone marrow samples from patients and stored them in anticoagulant ethylenediaminetetraacetic acid (EDTA) tubes until immunophenotyping was performed. The cell counts were done using a cytochemistry technique in an H1 autoanalyzer (Technicon, USA), and the number of leukocytes was adjusted to 5,000–10,000 cell isoton per microliter. Samples were stained directly using monoclonal tagged antibodies with fluorescence materials. Antibodies against human *CD135*-*PE*, *CD117-FITC* (BioLegend, USA), and white blood cells (WBCs) were gated and evaluated using the CD45-FITC index (Agilent DAKO, USA). In total, 5,000 events were acquired and the percent expressions of *CD135* and *CD117* on gated myeloblasts were recorded (Figure 1). The results were analyzed using the ProCell Quest software (BD, USA), and a threshold of 20% was taken to indicate cases positive for *CD117* and *CD135* expression Figure 1.

*Clinical outcome assessment*

After CR, all patients were examined every month for the first year and every 12 months thereafter. The OS and the EFS were the outcomes of interest: OS was defined as the time from diagnosis of AML to death from leukemia and/or the last follow-up time (December 2017); and EFS was defined as the time to the first event (remission, relapse, or death).

*Statistical analysis*

The baseline characteristics of patients were tested for normality using a test of skewness, and data were subsequently presented as mean (±SD) or as number and frequency, depending on the results. For categorization of quantitative values (including the expressions of *CD117*, *CD135*, and *CD135*+*CD117*), median values were calculated and used as cut-off points for categorized as high or low expression. We used the following cut-off points for other values: hemoglobin, <8 and ≥8 g/dL; WBC, <30,000 and ≥30,000 /mm^3^; and platelets, <30,000 and ≥30,000 /mm^3^.

The associations of *CD117* expression, *CD135* expression, and *CD135*+*CD117 *co-expression with other values were evaluated by Kruskal–Wallis tests and t-tests, using their mean values. For the survival analyses, we performed log-rank tests by the Kaplan–Meier method to assess the independent prognostic effects of *CD117*, *CD135*, and *CD135*+*CD117* expressions in patients with AML. We also performed Cox-proportional regression analyses to obtain the hazard ratios (HRs), standard errors, and 95% confidence intervals (CIs) of the prognostic factors. The prognostic index for each patient was calculated as the hazard coefficient of the three main values (hemoglobin, WBC, and *CD135*+*117*) multiplied by their scores. Log-rank tests and the Kaplan–Meier method were used to evaluate the significance of means of prognostic indexes on the OS and EFS. P-values ≤ 0.05 were considered statistically significant. All statistical analyses were done using STATA 11.0.

## Results

*Patient characteristics*

We enrolled 66 patients with AML in this study, among whom 42 (63.6%) were male and 24 (36.4%) were female and the mean age was 45.55 ± 12.21 years (range, 18–60 years). The baseline characteristics are shown in Table 1.

*Associations of patient characteristics with CD135 and CD117 expressions*

*CD135*, *CD117*, and *CD135*+*CD117* expressions on myoblasts were positive in 77.3%, 84.8%, and 68.2% of patients with AML, respectively; the corresponding median expressions were 72.5, 64.5, and 46.5, respectively (Table 2). There was a significant relationship between *CD135* expression and a higher mean WBC count (73.3% versus 47.9%, P = 0.002), but there were no significant relationships between other patient characteristics and the expressions of either* CD135* or *CD117* alone. The relationship between a lower mean hemoglobin and *CD135*+*CD117* co-expression approached significance (48.8% versus 35.4%, P = 0.08) (Table 3), but there were no significant relationships between any other patient characteristic and *CD135*+*CD117 *co-expression.

**Table 1:** Baseline Patient Characteristics

Variable	Positive	Median expression
*CD135*	51 (77.3%)	72.5
*CD117*	56 (84.8%)	64.5
*CD135*+*CD117*	45 (68.2%)	46.5

**Figure 2:**
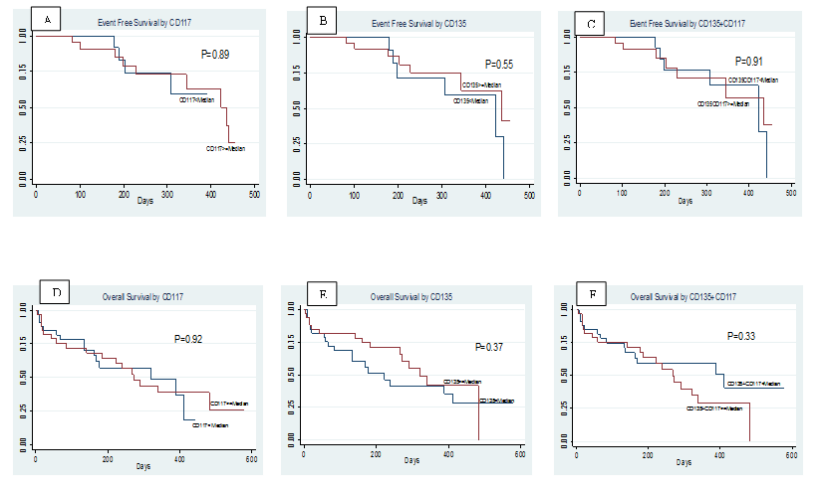
Kaplan-Meier EFS Curves for (A) *CD117*, (B) *CD135*, (C) Coexpression of *CD135* and *CD117*, and Kaplan-Meier OS Curves for (D)* CD117*, (E) *CD135*, (F) Coexpression of *CD135* and *CD117*

**Figure 3:**
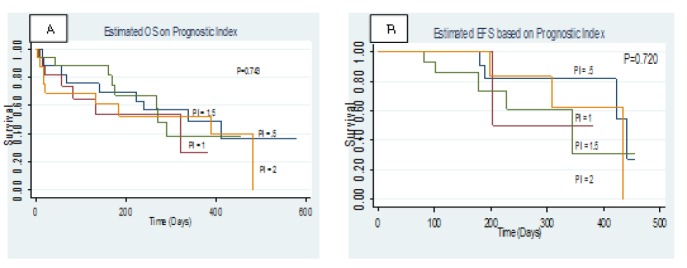
Difference in OS (A) and EFS (B) of the 4 Cohorts Based on Prognostic Index as Created by Hazard Coefficient of all the Significant Predictors for OS in Multivariable Analysis

**Table 2:** Expression of *CD135*, *CD117*, and *CD135*+*CD117*

Variables	Patients (N=66)
Median age in years (range)	45.55 (18 to 60)
Sex (male:female)	03:01
Median hemoglobin (g/dl) (range)	7.6 (3.4–14.1)
Median WBC (/mm^3^) (range)	14,645 (500–414,000)
Median Platelets (/mm^3^)	415,000 (6000-2, 74,000)
FAB subtype	
M0	4(6.1%)
M1	11(16.7%)
M2	34(51.5%)
M4	17(25.8%)

**Table 3:** Association of *CD135þCD117* Coexpression with Baseline Patient Characteristics

Variables	*CD135* (mean ±SD)	P 0.3	*CD117* (mean ±SD)	P 0.9	*CD135*+*CD117* (mean ±SD)	P 0.2
Sex						
Male (42)	55.9 ± 36.6		56.3 ± 27.7		39.7 ± 31.4	
Female (24)	63.6 ± 30.2		55.9 ± 30.0		48.4 ± 31.6	
Hemoglobin (g/dl)		0.1		0.1		0.08
<8 (37)	64.5 ± 31.1		60.4 ± 25.9		48.8 ± 30.0	
≥8 (29)	51.2 ± 37.3		50.6 ± 30.8		35.4 ± 32.3	
WBC (/mm^3^)		0.002		0.5		0.2
<30,000 (38)	47.9 ± 35.6		57.9 ± 30.8		39.1 ± 34.4	
≥30,000 (28)	73.3 ± 26.8		53.75 ± 25.0		48.0 ± 26.9	
Platelets (/mm^3^)		0.6		0.1		0.3
<30,000 (25)	56.2 ± 35.8		62.3 ± 25.0		47.7 ± 33.2	
≥30,000 (41)	60.2 ± 33.8		52.4 ± 29.9		40.0 ± 30.0	

**Table 4:** Univariate Survival Analysis for the *CD135*, *CD117*, and *CD135*+*CD117* Coexpression a. Mean /Days

Variables		CR %	P	EFS^a^ ± SE (CI)	P	OS^a^ ± SE (CI)	P
*CD117* (%)	< 64.5	14 (51.85)	0.82	326.57 ±26.25 (275.13- 378.01)	0.88	269.49 ±31.62 (207.51- 331.48)	0.92
	≥64.5	13 (48.15)		356.25 ±29.51 (298.41- 414.09)		303.68 ±41.44 (222.47- 384.90)	
*CD135* (%)	<72.5	12 (44.44)	0.50	347.86 ±37.21 (274.93- 420.78)	0.54	246.69 ±43.98 (190.49- 362.88)	0.37
	≥72.5	15 (55.56)		365.23 ±28.67 (309.03- 421.43)		309.82 ±34.83 (241.54- 378.09)	
*CD135*+*CD117* (%)	<46.5	16 (59.26)	0.18	361.70 ±32.40 (298.20- 425.21)	0.91	341.84 ±45.60 (252.47- 431.22)	0.33
	≥46.5	11 (40.74)		353.08 ±31.68 (290.99- 415.18)		263.01 ±33.78 (196.81- 329.22)	

**Table 5:** Multivariable Analysis with Significant Baseline Characteristics (Cox Proportional-Hazards Regression model)

		EFS				OS			
Variables		Hazard Ration	standard error	P	95% Confidence interval	Hazard Ration	standard error	P	95% Confidence interval
Hemoglobin (g/dl)	<8	-	-	-	- -	-	-	-	- -
	≥8	0.41	0.16	0.02	1.89 0.90	0.41	0.17	0.03	0.18 0.93
WBC (/mm^3^)	<30,000	1.76	0.68	0.14	0.82 3.77	1.68	0.63	0.17	0.80 3.54
	≥30,000	-	-	-	- -	-	-	-	- -
CD117 (%)	< 64.5	0.84	0.93	0.71	0.34 2.09	0.75	0.34	0.54	0.31 1.84
	≥64.5	-	-	-	- -	-	-	-	-
CD135 (%)	<72.5	0.34	0.16	0.02	0.13 0.88	0.36	0.17	0.03	0.14 0.93
	≥72.5	-	-	-	- -	-	-	-	- -
CD135 + CD117 (%)	<46.5	1.68	0.81	0.27	0.65 4.32	1.95	0.96	0.17	0.74 5.14
	≥46.5	-	-	-	- -	-	-	-	- -

*Survival analysis based on CD135 and CD117 expressions*

The mean follow-up time for OS was 197.83 ± 155.37 days. The OS was 50% and the mean OS was 303.72 days (range, 244.30–363.13 days), with a median of 290 days (range, 188.11–391.90 days). The mean follow-up time for EFS was 175.45 ± 136.47 days. The EFS was 80.3% and the mean EFS was 360.02 days (range, 317.36–402.67 days) with a median of 50.37 days (range, 323.29–520.72 days). *CD117*+*CD135* co-expression did not have a significant effect on either the OS (log-rank, P = 0.71) or the EFS (log-rank, p = 0.45). The CR rate was 40.9% overall, and at the median follow-up time of 168 days (range, 3–580 days), the OS and EFS were 73.98% ± 6.26% (CI: 59.31%–84.04%) and 47.61% ± 6.23% (95%CI, 35.05%–59.13%), respectively.

*The association between patient outcome and the expressions of CD135 and CD117*

In the univariate analyses, the expressions of *CD135*, *CD117*, and *CD135*+*117* were not significantly associated with the CR, EFS, or OS (Table 4). However, after adjustment for hemoglobin, WBC, *CD117*, *CD135*, and *CD117*+*CD135*, cox-regression analysis showed that a poor EFS was significantly associated with a high hemoglobin (HR 0.41, 95% CI 1.89–0.90, P = 0.027) and a low *CD135* (HR 0.34, 95% CI 0.13–0.88, P = 0.02). Cox-regression analysis also revealed that a poor OS was significantly associated with a high hemoglobin (HR 0.41, 95% CI 0.18–0.93, P = 0.03) and a low *CD135* (HR 0.36, 95% CI 0.14–0.93, P = 0.03). There were no significant associations between the EFS or the OS and either the mean WBC, the expression of *CD117*, or the co-expression of *CD135*+*117* (Table 5).

Based on the calculated coefficients, the following formula was used to calculate a prognostic index for each patient: (−0.81 × hemoglobin score) + (0.36 × WBC score) + (0.04 × *CD135*+*CD117* score). The median range of this prognostic index was then calculated and used to assess the OS (P = 0.74) and EFS (P = 0.72) at four different prognostic indexes (0.5, 1.0, 1.5, and 2.0), as shown in Figures 2 and 3.

## Discussion

AML is the most common leukemia in adults, yet it continues to have the lowest survival rate (Carow et al., 1996; Linnekin, 1999; Sharawat et al., 2015). The key proliferative RTKs in AML are CD117 and *CD135* (Sharawat et al., 2013) and whereas the role of *CD117* remains uncertain in hematopoietic neoplasia, the *CD135* antigen is recognized as the FLT3 ligand of its receptor-signaling pathway. Although it is rarely tested in acute leukemia (Noronha et al., 2016; Paietta et al., 2004) a few studies have found that the *CD135* antigen is highly expressed in B-cell ALL, in B-cell lines, in AML, and to a lesser degree in T-cell ALL (Drexler, 1996; Noronha et al., 2016; Wells et al., 1996). In the present study, the expressions of *CD135*, *CD117*, and *CD135*+*CD117 *were explored in 66 patients with AML, and they were shown to be expressed on the myoblasts of 77.3%, 84.8%, and 68.2% of patients with AML, respectively (Table 2). This is consistent with research by Wells et al., (1996) where *CD117* was present in 87% of cases of AML, and with the results of a cohort study by Sharawat et al., (2013), where *CD135* and *CD117* expressions >20% were observed in 82% and 90% of patients’ myeloblasts.Tarlock et al., (2017) reported that there was no significant associa-tion between *CD135* expression and the WBC count. In our study, however, there was a significant relationship between *CD135 *expression and a higher mean WBC count (73.3% versus 47.9%, P = 0.002), although we cannot exclude the possibility that this was caused by our small sample. We could find no associations between other patient characteristics and the expressions of *CD135*, *CD117*, or *CD135*+*CD117*. The co-expression of CD135+CD117 has been discussed by Wells et al., (1996) but there have been no reports of how they relate to the OS and EFS. The co-expression of CD135+CD117 did not significantly affect the OS (log-rank, P = 0.71) or the EFS (log-rank, p = 0.45) in our study. However, in a cohort study by Sharawat et al., (2013), univariate analysis indicated that high expression of* CD135* and high co-expression of *CD135*+*CD117* was significantly associated with the CR rate (P < 0.001 and P < 0.006, respectively). We found no such relationship, which again was probably due to the small sample size. Multivariable analysis in the study by Sharawat et al., (2013). Also showed that the expressions of *CD135* or *CD117* alone did not predict survival, but it did show that their co-expression was the strongest independent predictor of both EFS (HR 2.46) and OS (HR 2.25). Our cox-regression analysis revealed that poor EFS and OS rates were significantly associated with high hemoglobin levels and low *CD135* expression (Table 5).

This study represents the first in Iran to show a correlation between RTKs (*CD135* and *CD117*) and poor survival in patients with AML. However, this study is not without limitations. We only analyzed a small number of cases and cannot exclude the possibility of selection bias. Also, the study had a short follow-up period for determining survival compared with other studies on this topic, making it difficult to assess the full impact of *CD135*+*CD117* co-expression on disease progression and death. Despite these limitations, our study benefits from having evaluated the expressions of* CD117*,* CD135*, and *CD135*+*CD117* in patients with AML in the context of the EFS and OS. This is important because clinical trials have used FLT3 antagonists therapeutically, and one of the most famous ones involved using c-KIT inhibitors in patients with elevated *CD135* and *CD117* expressions (Sharawat et al., 2013). This remains a promising area for future research and treatment.

In conclusion, in summary, we have shown that *CD135 *and *CD117* co-expression was observed in 68.2% of patients with AML in our cohort in Iran. This expression is easily measured by conventional diagnostic flowcytometry techniques and could be used as an independent prognostic marker of AML. Considering the results of this study, a large study is needed to assess the efficacy of the identified markers included in our prognostic index (i.e., WBC count, hemoglobin level, and the percentage co-expression of *CD135 *and *CD117*).
